# Levels and Predictors of Knowledge, Attitudes, and Practices Regarding Contraception Among Female TV Studies Undergraduates in Nigeria: Cross-Sectional Study

**DOI:** 10.2196/56135

**Published:** 2025-05-08

**Authors:** Hadizah Abigail Agbo, Philip Adewale Adeoye, Danjuma Ropzak Yilzung, Jawa Samson Mangut, Paul Friday Ogbada

**Affiliations:** 1 Department of Community Medicine, Jos University Teaching Hospital, Lamingo, Jos, Plateau State, Nigeria, +234 8062081617; 2 Department of Community Medicine, University of Jos, Jos, Nigeria; 3 College of Health Sciences, University of Jos, Jos, Nigeria

**Keywords:** knowledge, attitudes, practice, contraception, regression, cross-sectional, female, students, Nigeria

## Abstract

**Background:**

Access to contraception is a preventive measure against unplanned pregnancy and sexually transmitted infections; especially in sub-Saharan Africa where unmet need is a public health concern.

**Objective:**

This study assessed the levels and predictors of knowledge, attitudes, and practices regarding contraception among female TV studies students in Nigeria.

**Methods:**

This is a cross-sectional study conducted among female students of NTA TV College, Nigeria. Categorical sociodemographics, knowledge, attitude, and practice were presented as frequencies and proportions, while the continuous variables were presented as summary measures of central tendencies and dispersions. The primary outcome variable was the practices regarding contraception, while attitude and knowledge were secondary outcome variables, with sociodemographics as covariates. Predictors of good knowledge, attitude, and practice regarding contraception were determined by multivariable binary logistic regression, which was preceded by a bivariate regression analysis to determine candidate variables for the final model. A *P* value <.05 was determined to be statistically significant.

**Results:**

There were 217 study participants with an average age of 22 (SD 2.6) years. Levels of good knowledge, attitude, and practice regarding contraception were reported in 55.3% (n=120), 47.5% (n=103), and 50.7% (n=110) of participants, respectively. The majority have had sex, used friends and the internet as their main sources of contraceptive information, and commonly used contraceptives such as condoms and oral contraceptive pills. The most common reason for not using contraceptives was fear of side effects or health risks. Being a young adult was a significant predictor (adjusted odds ratio [aOR] 2.6, 95% CI 1.0‐6.7; *P*=.04) of good knowledge, while being a diploma student (aOR 2.4, 95% CI 1.2‐4.6; *P*=.01), living off campus (aOR 2.1, 95% CI 1.0‐4.4; *P*=.04), and good knowledge (aOR 3.8, 95% CI 2.1‐6.9; *P*<.001) were significant predictors of good attitude. Being from the state’s indigenous population (aOR 2.4, 95% CI 1.2‐4.6; *P*=.01) and having engaged in sex (aOR 24.5, 95% CI 7.9‐75.7; *P*<.001) were significant predictors of good contraception use.

**Conclusions:**

Our study has shown relatively low levels of good knowledge, attitude, and practice regarding contraception and their predictors. Therefore, there is an urgent need to consistently improve advocacy, curricular development, and policies to improve knowledge, attitude, and practice regarding contraception and sexual and reproductive health services among young people.

## Introduction

Worldwide, the proportion of women with unmet needs for modern contraception is highest in sub-Saharan Africa—twice the world’s average [1]. This unmet need reportedly leads to unwanted pregnancies, unsafe abortions, and the limited ability of women to advance educationally, career-wise, and economically. The use of contraceptives should be a right-based issue that is necessary for ensuring informed choices regarding family planning. Correct use can significantly improve women’s reproductive health and well-being [2]. Contraception can be an important measure against unintended pregnancy, abortion, and sexually transmitted infections (STIs), especially among young people. Recent efforts in the last decade have sought to reduce unmet needs among women and girls [3].

The proportion of youth (aged 15‐35 years) in Nigeria is reported to be about half of the population, with about 57% who have never married [4]. This group constitutes the highest proportion of those who join the higher education system yearly. Though this group is the most sexually active and has higher contraceptive use rates, they also have the highest level of unmet needs among all population groups [5-8]. They are more likely to have premarital sex, often without protection; early, multiple, and short-lived intimate relationships; limited knowledge of sexuality issues that are required for a healthy sex life and reduction in the risk of teenage pregnancy, unsafe abortions, and STIs; and less likely to discuss family planning issues with health care providers [6,8], which poses public health and social problems in many lower- and middle-income countries, with many studies indicating increasing incidence of unsafe abortion, STIs including HIV/AIDS, violence against girls, and pregnancy-related morbidity and mortality [8-10]. Unintended and unwanted pregnancy among university students may jeopardize their academic pursuit and potential future careers.

Despite the increased accessibility of contraceptives at health facilities across the country, use remains low among young females. General uptake varies across the region, with the North Central region having the most unmet needs. Girls faced more challenges accessing contraceptives than women living with intimate partners due to the associated stigma associated with their premarital sexual activities [1,6,8,11]. Additionally, they are faced with limited access to health-related information about contraception, with regional variations, with their perception being guided by the prevailing sociocultural norms and peer influence [1,6,8]. Thus, young people lack the needed self-efficacy to negotiate a healthy sexual encounter.

Several studies have reported contraceptive knowledge, attitude, and practice among various groups of young people. Recent reports have shown that exposure to mass media communication regarding family planning increases the likelihood of use in sub-Saharan Africa [12]. With Nigeria’s high fertility rate and a corresponding maternal mortality rate [6,13,14], efforts should be geared at increasing and monitoring access to family planning information and services among young women to ensure a healthy reproductive life and general well-being.

Unfortunately, there is currently a curricular deficit in the training of TV/broadcast undergraduates on health communication in Nigeria, which will continue to perpetuate professional incapacitation of future practitioners, such as limited knowledge of health issues, and the interpretation and contextualization of such to a target audience [15]. Exposure to mass media has been shown as a proven means of improving knowledge, positive attitude, advocacy, and self-efficacy for contraception use. Appropriate, adequate, and consistent training of TV producers, anchors, and writers, and other creative writers will ensure accurate and reliable health information dissemination and improve reproductive health outcomes and accountability [16].

Additionally, little is known about contraceptive knowledge, attitude, and practice among tertiary education students in mass communication, journalism, and TV studies disciplines, who may be involved in the conceptualization, development, and implementation of information, communication, and education programs, and mass media campaign activities regarding contraception among communities and the nation in the future. The state where the study was done has been shown to have the highest level of unmet needs in Nigeria’s North Central region among married females [6]; therefore, there is a need to assess contraceptive behavior among the study population. This study assessed the level and predictors of contraception knowledge, attitudes, and practices of female TV studies students in Nigeria.

## Methods

### Overview

This is a cross-sectional study. The conceptual theory for this study is the health belief model. There are 7 constructs in the model, which were applied to the use of contraceptives among the study participants. Perceived susceptibility to unwanted pregnancy may make an individual evaluate the perceived severity or consequences of unintended pregnancy. This may drive the individual to evaluate the perceived benefits of contraceptive use to prevent the perceived threat of contraceptive nonuse. Perceived barriers to contraceptive use will be thoroughly evaluated to decide on the feasibility of contraceptive use. However, because human behavioral change takes time, there will be the need to remind individuals to adopt and maintain contraceptive use often (cues to action) via mass media, information and communication technology, family and peers, and the development of self-efficacy for contraceptive use [17].

### Study Area

Plateau State is located in the North Central part of Nigeria. It covers a land area of 26,899 km^2^, with an estimated population of about 4.9 million [18]. It has over 40 ethnolinguistic groups, and each group has its distinct language. English and Hausa are common spoken languages in Plateau State. It is bounded in the northeast by Bauchi State, the northwest by Kaduna, the southwest by Nasarawa, and the southeast by Taraba State. Though situated in a tropical area, it has a near-temperate climate due to its high altitude. It has 17 local governments of which Jos North, Jos South, and Jos East make up the Jos metropolis. It has 12 higher institutions awarding various postsecondary school certificates, among which are 5 specialized educational institutions including NTA TV College [19].

NTA TV College was created in 1980 to meet the need to train the TV workforce to meet broadcast challenges in Nigeria. It is located in Rayfield, Jos South Local Government Area, Jos, Plateau State. From the beginning, it was concerned with the conduct of continuous professional development and short courses for TV industry stakeholders. It was later upgraded to offer diploma and degree programs. Nigeria’s only higher institution of TV studies is currently affiliated with Ahmadu Bello University, Zaria-Nigeria, to offer a mass communication degree in TV production and journalism [20].

### Study Population

The study population was female students of NTA TV College who gave their consent to participate in the study. Female students of NTA TV College who withdrew their consent to participate at any stage of the study and those who were not available for one reason or the other during the research were excluded from the study.

### Sample Size Determination

Calculation of the sample size was determined using the Cochran sample size determination formula [21], mathematically expressed as:

n = z²pq/d²

Where n is the minimum sample size, z is the standard normal deviate at a 95% CI (1.96), p is the proportion of female undergraduates who are aware of contraception (0.84) [22], q is the alternate probability (1 – 0.84 = 0.16), and d is the precision (5%=0.05). Therefore, 206 was the estimated sample size. After adjusting for 10% nonresponse, the estimated sample size was 227.

### Sampling Technique

The female students were selected using a simple random technique by balloting from the six levels during classes in the college using a proportionate approach. There were about 1021 students at the time of the study, with 220 in Ordinary National Diploma (OND) 1 (n=47, 21.7% of final sample), 208 in OND 2 (n=44, 20.3% of final sample), 29 in 100L (n=6, 2.8% of final sample), 184 in 200L (n=39, 18% of the final sample), 215 in 300L (n=46, 21.2% of the final sample), and 165 in 400L (n=35, 16.1% of final sample).

### Study Instrument and Data Collection Methods

Data were collected from the female students of NTA TV College by the research team using a semistructured self-administered questionnaire after informed written consent was obtained (Multimedia Appendix 1). The questionnaire is divided into four sections: social demographic characteristics, knowledge of contraception, attitudes toward contraceptives, contraceptive practices, and sexual behavior. The questionnaire was pretested and pilot-tested among female students at the National Film Institute Jos, which has a comparable population to our study population, to identify errors, test the fidelity of the research process and the feasibility of the study, and observe the understandability of the research tools by the research participants during the pilot.

### Data Management and Analysis

The data obtained were entered and analyzed using SPSS (version 25; IBM Corp). Qualitative data such as sex and religion were presented using frequencies and percentages, while quantitative data were presented using means and SDs, except when not normally distributed. Age was categorized as adolescents (≤19 years), according to the World Health Organization and published literature [8,23], and young adults (>19 years), according to the classification of the study population by Statistics Canada [24]. The average scores were used to compute the levels of contraception knowledge, attitudes, and practice, with scores less than the average classified as poor and those equal to or greater than the average score classified as good. The primary outcome variable is contraception practices, while attitude and knowledge were secondary outcome variables. Sociodemographics, knowledge, and attitude served as covariates or independent variables, as applicable. Simple logistic regression was used to determine the factor that is associated with contraception knowledge, attitudes, and practices, and to determine the candidate variables for the multivariable analysis. Variables with less than 10% probability were selected and added to the omnibus model to determine the predictors of contraception knowledge, attitudes, and practices. A *P* value <.05 was considered significant. Model characteristics and fitness for each multivariable logistic regression are stated with each result.

### Ethical Considerations

Ethical clearance was obtained from the Jos University Teaching Hospital Research and Ethics Committee (JUTH/DCS/IREC/127/XXXI/2619). Written informed consent was obtained before participation, which was voluntary, and clients were free to withdraw consent at any point. There was no potential hazard to the study participants that might warrant exclusion or treatment. Data collection was self-administered to prevent interference by a third party when there was no other person to ensure privacy. No identification entries (name, phone numbers) were allowed. All study participants were assured that the data would be used for academic and research purposes only. No compensation was given, except for the health education on contraception after the final data collection.

## Results

There was a 95.6% (217/227) response rate among study participants.

Table 1 shows the sociodemographic characteristics of the 217 participants. The majority were young adults, with an average age of 21 (range 17-32) years. The majority were either single or separated (n=198, 91.3%), with singles being 90.8% (n=197) and separated respondents being 0.5% (n=1) of the total study population. The majority were of the Christian faith, and a little more than 10% were of the Islamic faith. Though more students were out of state compared to their ethnic origin, they were mostly in-state residents. More students were in degree programs and earned less than the minimum wage. More than two-thirds were TV journalism students, and more than three-quarters lived off campus.

**Table 1.** Sociodemographic characteristics of study participants (n=217).

Variable	Values
Age (years), mean (SD)	21.9 (2.6)
≤19 years (adolescents), n (%)	34 (15.7)
>19 years (young adults), n (%)	183 (84.3)
Marital status, n (%)	
Single/separated	198 (91.3)
Married	19 (8.7)
Religion, n (%)	
Christianity	186 (85.7)
Islam	31 (14.3)
Tribe/ethnicity, n (%)	
Plateau indigenous	91 (41.9)
Plateau nonindigenous	126 (58.1)
Program, n (%)	
Diploma	91 (41.9)
Degree	126 (58.1)
Level in school, n (%)	
OND1^a^/OND2 (early classes)	91 (41.9)
100L/200L (middle classes)	45 (20.7)
300L/400L (older classes)	81 (37.3)
Monthly income^b^ (n=149), median (IQR)	₦17,500 (₦10,000-₦23,000)
<₦18,000, n (%)	76 (51.0)
≥₦18,000, n (%)	73 (49.0)
Home residence (n=201), n (%)	
Plateau State	126 (62.7)
Outside Plateau State	75 (37.3)
School residence (n=215), n (%)	
Campus	50 (23.3)
Off campus	165 (76.7)
Department (n=214), n (%)	
TV journalism	149 (69.6)
TV production	65 (30.4)

a OND: Ordinary National Diploma.

b A currency exchange rate of US $1=₦415 is applicable as of February 18, 2022.

Table 2 shows the level of contraception knowledge, attitudes, and practices among study participants. The classification was based on the use of average scores, with the good class having at least the average score and the poor class having less than the average score. It shows that just above half reported good knowledge, attitude, and practices. The average score of contraception knowledge, attitudes, and practices were 50%, 71.3%, and 35%, respectively. Almost three-quarters (160/217, 73.7%) have had sexual intercourse.

Table 3 shows the sources of information on contraception among the participants. It shows that friends (83/236, 35.2%) and the internet (81/236, 34.3%) were the most common sources of information on contraception (with 43/236, 18.2% using Google search and 38/236, 16.1% accessing contraceptive information via social media), which was followed by family. The least used sources were newspapers and magazines.

**Table 2.** Level of knowledge on, attitudes toward, and practices of contraception and engagement in sexual activity among study respondents (N=217).

Variables	Values
Knowledge level, n (%)	
Poor knowledge	97 (44.7)
Good knowledge	120 (55.3)
Average knowledge score (%), median (IQR)	50.0 (33.0-58.0)
Attitude level, n (%)	
Good attitude	103 (47.5)
Poor attitude	114 (52.5)
Average attitude score (%), mean (SD)	71.3 (10.6)
Practice level, n (%)	
Poor practice	107 (49.3)
Good practice	110 (50.7)
Average practice score (%), median (IQR)	35.0 (28.0-52.0)
Sexual behavior, n (%)	
Ever had sexual intercourse	160 (73.7)
Never had sexual intercourse	57 (26.3)

**Table 3.** Sources of information on contraception among study respondents (n=236).

Source^a^ of contraception information	Responses, n (%)	
Family	29 (12.3)	
Friends	83 (35.2)	
Print media newspaper	8 (3.4)	
Print media magazines	7 (3.0)	
Internet: Google	43 (18.2)	
Internet: social media	38 (16.1)	
Broadcast media TV	20 (8.5)	
Health facility	8 (3.4)	

a Participants could pick more than one source of contraceptive information, and a multiple-response analysis was done.

Table 4 shows the specific contractive methods currently being used or that were ever used among study participants. Only 85 of the 217 (39.2%) respondents disclosed the specific contraception being used. It shows that condoms (37/85, 44%) and oral contraceptive pills (OCPs; 31/85, 36%) were the most common contraceptives used by students at NTA TV College. Others, which accounted for 4.7%, have used implants, emergency contraception (EC), and other unnamed forms of contraception.

**Table 4.** Specific contraceptives currently being used or ever used among study respondents (n=85).

Variables	Responses, n (%)	
Condom	37 (44)	
Oral contraceptive pill	31 (36)	
IUCD^a^	1 (1)	
Injectable	3 (4)	
Withdrawal	2 (2)	
Calendar method	5 (6)	
Billings	2 (2)	
Others	4 (5)	

a IUCD: intrauterine contraceptive device.

Table 5 shows the reasons why respondents do not use contraceptives. It shows that the most common reason why respondents will not use contraceptives is because of side effects, distantly followed by the perception that it increases the risk of health issues and because they are single.

**Table 5.** Reasons why respondents will not use contraceptives among study respondents (n=118)^a^.

Response	Responses, n (%)	
Accessibility	1 (0.8)	
Based on the objection of the partner	5 (4.2)	
Because I am single	11 (9.3)	
Because I don’t intend on having sex	6 (5.1)	
Because I don’t need it	5 (4.2)	
Because of the side effect	35 (29.7)	
Contraceptives sometimes are not 100% guarantee	3 (2.5)	
Cultural believe	2 (1.7)	
Delays pregancy	8 (6.8)	
Don’t need it	7 (5.9)	
I don’t know	6 (5.1)	
I have used it before	2 (1.7)	
I love my life	1 (0.8)	
I need child	4 (3.4)	
It damages the womb	5 (4.2)	
It depends on your fertility	1 (0.8)	
It increase the risk of health issues	12 (10.2)	
It makes people gain weight	1 (0.8)	
Religious belief	2 (1.7)	
I calculate my fertile days	1 (0.8)	

a Participants could pick more than one source of contraceptive information, and a multiple-response analysis was done.

Table 6 shows the predictors of contraception knowledge among study participants with the model characteristics of the multivariable logistic regression. In the bivariate analysis, being a young adult (odds ratio 3.6, 95% CI 1.6-8.0) was associated with good knowledge compared to being an adolescent in the study population. Additionally, being in the middle classes (100L/200L) was associated with good contraception knowledge compared to those in the older classes (300L/400L). The multivariable logistic regression showed that being a young adult (aged >19 years) was a significant predictor of good knowledge of contraception (adjusted odds ratio [aOR] 2.6, 95% CI 1.0-6.7; *P*=.04) compared to being an adolescent (aged ≤19 years) among the study population.

**Table 6.** Predictors of contraception knowledge among study respondents.^a^

Variable	*β*	OR^b^ (95% CI)	*P* value	*β*	aOR^c^ (95% CI)	*P* value
Age						
>19 years (young adults)	1.3	3.6 (1.6‐8.0)	.002^d^^,^^e^	1.0	2.6 (1.0‐6.7)	.04^d^
≤19 years (teenagers; reference)	—^f^	1	—	—	1	—
Marital status						
Single/separated	0.6	1.8 (0.7‐4.6)	.23	—	—	—
Married (reference)	—	1	—	—	—	—
Religion						
Islam	0.1	1.1 (0.5‐2.5)	.74	—	—	—
Christianity (reference)	—	1	—	—	—	—
Tribe/ethnicity						
Plateau indigenous	0.3	1.3 (0.8‐2.3)	.31	—	—	—
Plateau nonindigenous (reference)	—	1	—	—	—	—
Program						
Degree	0.2	1.2 (0.7‐2.1)	.52	—	—	—
Diploma (reference)	—	1	—	—	—	—
Level in school						
OND1^g^/OND2 (early classes)	0.1	1.1 (0.6‐2.0)	.78	—	—	—
100L/200L (middle classes)	0.8	2.2 (1.0‐4.7)	.049^d^	—	—	—
300L/400L (older classes; reference)	—	1	—	—	—	—
Monthly income^h^						
≥₦18,000	0.6	1.8 (0.9‐3.5)	.08^d^^,^^e^	0.5	1.7 (0.9‐3.3)	.11
<₦18,000 (reference)	—	1	—	—	1	—
Home residence						
Outside Plateau State	0.1	1.0 (0.6‐1.8)	.98	—	—	—
Plateau State (reference)	—	1	—	—	—	—
School residence						
Off campus	0.3	1.3 (0.7‐2.5)	.97	—	—	—
Campus (reference)	—	1	—	—	—	—
Department						
TV journalism	0.1	1.0 (0.6‐1.8)	.97	—	—	—
TV production (reference)	—	1	—	—	—	—

a Model characteristics: –2log likelihood 197.207, Cos & Snell *R*2=0.048, Nagelkerke *R*2=0.065, Hosmer-Lemeshow *P*=.22, overall percentage accuracy 60.4%.

b OR: odds ratio.

c aOR: adjusted odds ratio.

d Significant at *P*<.05.

e Candidate variables for multiple log regression at *P*<.10.

f Not applicable.

g OND: Ordinary National Diploma.

h A currency exchange rate of US $1=₦415 is applicable as of February 18, 2022.

Table 7 shows the predictors of attitude toward contraception and the model characteristics of multivariable logistic regression. It shows that, in the bivariate analysis, being in the older and middle classes was associated with less likelihood of a good attitude toward contraception. Additionally, staying in an off-campus residence was associated with twice as higher likelihood of having a good attitude toward contraception. Good knowledge was associated with a 3.5 higher likelihood of a good attitude toward contraception among the study population. In multivariable logistic regression, being a diploma student (aOR 2.4, 95% CI 1.2-4.6; *P*=.01) was a significant predictor of a good attitude toward contraception compared to those in degree programs, having an off-campus accommodation at school was a significant predictor of good attitude (aOR 2.1, 95% CI 1.0‐4.4; *P*=.04) compared to those with on-campus accommodations, and having good knowledge was a significant predictor of good attitude (aOR 3.8, 95% CI 2.1‐6.9; *P*<.001) compared to those with poor knowledge.

**Table 7.** Predictors of attitude toward contraception among study respondents.^a^

Variable	*β*	OR^b^ (95% CI)	*P* value	*β*	aOR^c^ (95% CI)	*P* value
Age						
≤19 years (teenagers)	0.4	1.5 (0.7‐3.1)	.29	—^d^	—	—
>19 years (young adults; reference)	—	1	—	—	—	—
Marital status						
Single/separated	0.5	1.6 (0.6‐4.3)	.34	—	—	—
Married (reference)	—	1	—	—	—	—
Religion						
Islam	0.2	1.2 (0.6‐2.6)	.62	—	—	—
Christianity (reference)	—	1	—	—	—	—
Tribe/ethnicity						
Plateau indigenous	0.1	1.1 (0.7‐2.0)	.62	—	—	—
Plateau nonindigenous (reference)	—	1	—	—	—	—
Program						
Diploma	0.8	2.1 (1.2‐3.7)	.007^e^^,^^f^	0.9	2.4 (1.2‐4.6)	.01^e^
Degree (reference)	—	1	—	—	1	—
Level in school						
300L/400L (older classes)	–0.7	0.5 (0.3‐0.9)	.02^e^^,^^f^	—	—	—
100L/200L (middle classes)	–0.8	0.4 (0.2‐0.9)	.02^e^^,^^f^	–0.3	0.8 (0.3‐1.7)	.52
OND1^g^/OND2 (early classes; reference)	—	1	—	—	1	—
Monthly income^h^						
≥₦18,000	0.5	1.6 (0.8‐3.0)	.17	—	—	—
<₦18,000 (reference)	—	1	—	—	—	—
Home residence						
Plateau State	0.1	1.1 (0.6‐2.0)	.67	—	—	—
Outside Plateau State (reference)	—	1	—	—	—	—
School residence						
Off campus	0.7	2.1 (1.1‐4.0)	.03^e^^,^^f^	0.8	2.1 (1.0‐4.4)	.04^e^
Campus (reference)	—	1	—	—	1	—
Department						
TV journalism	0.1	1.1 (0.6‐1.9)	.84	—	—	—
TV production (reference)	—	1	—	—	—	—
Level of knowledge						
Good knowledge	1.2	3.5 (2.0‐6.1)	<.001^e^^,^^f^	1.3	3.8 (2.1‐6.9)	<.001^e^
Poor knowledge (reference)	—	1	—	—	1	—

a Model characteristics: –2log likelihood 264.244, Cos & Snell *R*2=0.143, Nagelkerke *R*2=0.191, Hosmer-Lemeshow *P*=.90, overall percentage accuracy 67.9%.

b OR: odds ratio.

c aOR: adjusted odds ratio.

d Not applicable.

e Significant at *P*<.05.

f Candidate variables for multiple logistic regression at *P*<.10.

g OND: Ordinary National Diploma.

h A currency exchange rate of US $1=₦415 is applicable as of February 18, 2022.

Table 8 shows the predictors of contraceptive practice among the study population. In the bivariate analysis, being in the middle class was associated with a 2.5 higher likelihood of good contraceptive practice. Good knowledge of contraception was associated with a 2.5 higher likelihood of good contraceptive practice. Good attitude was associated with a twice higher likelihood of good contraceptive practice, and having engaged in sex was associated with good contraceptive practice. In the multivariable logistic regression, being from the state’s indigenous (majority) population was a significant predictor of good contraceptive practice (aOR 2.4, 95% CI 1.2‐4.6; *P*=.01) compared to those from the nonindigenous population. Having engaged in sex (aOR 24.5, 95% CI 7.9-75.7; *P*<.001) was a significant predictor of good contraceptive practice compared to those who had never engaged in sex.

**Table 8.** Predictors of the practice of contraception among study respondents.^a^

Variable	*β*	OR^b^ (95% CI)	*P* value	*β*	aOR^c^ (95% CI)	*P* value
Age						
>19 years (young adults)	0.7	2.1 (1.0‐4.5)	.05^d^	–0.4	0.7 (0.2‐2.1)	.51
≤19 years (teenagers; reference)	—^e^	1	—	—	1	—
Marital status						
Married	0.1	1.1 (0.4‐2.8)	.86	—	—	—
Single/separated (reference)	—	1	—	—	—	—
Religion						
Christianity	0.1	1.1 (0.5‐2.4)	.78	—	—	—
Islam (reference)	—	1	—	—	—	—
Tribe/ethnicity						
Plateau indigenous	1.0	2.7 (1.6‐4.7)	<.001^d^^,^^f^	0.9	2.4 (1.2‐4.6)	.01^f^
Plateau nonindigenous (reference)	—	1	—	—	1	—
Program						
Degree	0.2	1.2 (0.7‐2.0)	.56	—	—	—
Diploma (reference)	—	1	—	—	—	—
Level in school						
OND1^g^/OND2 (early classes)	0.2	1.2 (0.6‐2.1)	.61	—	—	—
100L/200L (middle classes)	0.9	2.5 (1.2‐5.3)	.02^d^^,^^f^	—	—	—
300L/400L (older classes; reference)	—	1	—	—	—	—
Monthly income^h^						
≥₦18,000	0.2	1.2 (0.6‐2.3)	.56	—	—	—
<₦18,000 (reference)	—	1	—	—	—	—
Home residence						
Plateau State	0.4	1.5 (0.9‐2.7)	.15	—	—	—
Outside Plateau State (reference)	—	1	—	—	—	—
School residence						
Campus	0.1	1.1 (0.6‐2.0)	.83	—	—	—
Off campus (reference)	—	1	—	—	—	—
Department						
TV journalism	0.3	1.4 (0.8‐2.5)	.26	—	—	—
TV production (reference)	—	1	—	—	—	—
Level of knowledge						
Good knowledge	0.9	2.5 (1.5‐4.4)	.001^d^^,^^f^	0.6	1.8 (0.9‐3.7)	.09
Poor knowledge (reference)	—	1	—	—	1	—
Attitude						
Good attitude	0.7	2.1 (1.2‐3.6)	.008^d^^,^^f^	0.5	1.6 (0.8‐3.1)	.20
Poor attitude (reference)	—	1	—	—	1	—
Sexual behavior						
Ever	3.3	26.0 (8.9‐75.7)	<.001^d^^,^^f^	3.2	24.5 (7.9‐75.7)	<.001^f^
Never (reference)	—	1	—	—	1	—

a Model characteristics: –2log likelihood 216.338, Cos & Snell *R*2=0.322, Nagelkerke *R*2=0.43, Hosmer-Lemeshow *P*=.99, overall percentage accuracy 75.6%.

b OR: odds ratio.

c aOR: adjusted odds ratio.

d Candidate variables for multiple log regression at *P*<.10.

e Not applicable.

f Significant at *P*<.05.

g OND: Ordinary National Diploma.

h A currency exchange rate of US $1=₦415 is applicable as of February 18, 2022.

## Discussion

Our study shows that about half of all respondents had good knowledge, attitudes, and practices regarding contraception, with almost three-quarters having had sex and their main sources of contraceptive information being friends and the internet. Commonly used contraceptives were condoms and OCPs. A common reason for the nonuse of contraceptives was fear of side effects or health risks. Age was observed to be a significant predictor of good knowledge of contraception, while being in a diploma program (lower degree), living off campus, and having good knowledge were significant predictors of a good attitude toward contraception. Ethnicity and sexual behavior were significant predictors of good contraception use.

Our study revealed that about half of the respondents had good knowledge. This is similar to a study in Botswana [25]. However, a lower level of good knowledge was observed among students from Selangor, Malaysia; Spain; Imo State, Nigeria; and Ethiopia [26-29], while a higher level of good knowledge was observed among students in Dodoma, Tanzania; Kano and South-South, Nigeria; Pretoria, South Africa; and Kwadaso, Ghana [30-34]. These results support the evidence that one of the major professional issues in health broadcasting and programming in Nigeria is a lack of deep specialized knowledge in health communication and programming [15]. Better knowledge among this student population will improve confidence in reporting and programming, improve demand for accountability from stakeholders, increase journalist-led family planning stories and programming, and generally raise awareness in communities about issues related to contraception and general health [16].

This study also revealed that almost half of the respondents had a good attitude toward contraception. This is similar to studies in Selangor, Malaysia and the emerging region of Ethiopia [26,35]. However, higher levels of good attitude were seen in Kano, Nigeria; Adama and the emerging regions of Ethiopia; Pretoria, South Africa; Kwadaso, Ghana; and Spain [27,31,33-35]. Since health broadcast professionals cannot be said to be unattached, uninvolved, unbiased, and dispassionate in the production and transmission of content, their attitudes, philosophies, beliefs, and feelings might shape their approach, strategies, language choice, and angle for relaying health messages [15,16]. Counteracting negative attitudes and stereotypes should be sustained through community dialogues (while in school and during their professional life) to improve the interest of future information professionals in issues relating to family planning and general health.

It was observed that half of the study participants had good contraceptive practices. This is lower than reported among students in Kano, Nigeria [31]. This may be due to the recent 5-state public-private partnership geared toward increasing contraceptive uptake, of which Kano is a part. There was also a financially higher commitment to family planning services in these states compared to Plateau State [36,37]. A recent report of the 5-state intervention revealed increased demand generation and uptake, and improved state government financing of contraception services [36]. This government-nongovernmental organization effort might have rubbed off on young female college students in Kano. Additionally, the recent Nigerian Demographic and Health Survey reported that women of reproductive age in Kano reported a higher level of exposure to family planning messages and discussion with health care workers on family planning during their visits to health facilities compared to Plateau women of reproductive age [6]. When health communicators are also good practitioners of their message, it increases positive decision-making among target populations. Thus, public-private initiatives that engage current and future health broadcasters and program officers to improve and sustain the current gains of contraceptive uptake should be encouraged among young people [16].

Almost three-quarters of our sample had sex. This is similar to the sexual behavior seen among students in South-South, Nigeria [32]; lower compared to students in Spain [27]; and higher than those reported among similar populations in Botswana, urban Nigerian cities, and Kilimanjaro Region of Tanzania [7,25,38,39]. This may be a result of an increased liberal worldview among young people, a sense of freedom, and a desire for sexual experimentation in the university environment. There is a need for early sexual and reproductive education to empower young people against risky sexual exploitation and behaviors.

Friends and the internet were the most common sources of information on contraception. This is similar to studies from Kilimanjaro, Tanzania; Botswana; Ilorin and South-South, Nigeria; Dodoma, Tanzania; and Spain among students [25,27,30,38,40]. However, health facilities and health care workers were the most common sources of information on contraception among similar populations in Kwadaso, Ghana and the emerging regions of Ethiopia [34,35]. Information on contraception from family members often comes out of concern that a young person is sexually or about to be sexually active, and therefore knowledge of safe sex is needed. Family members, sisters, and mothers are highly trusted based on their overall familial relationship. Additionally, trust in internet sources was often improved among young women when the source of the information is from reputable sites such as those indicating .org, .edu, and .gov [41]. Therefore, such sites should be protected from being hacked or contaminated by conspiracy theories, overt political commentaries, and unscientific content. Limited access to health information and family planning messages might be due to inadequate health broadcasting scheduling and programming. The lack of dedicated health broadcast stations and barriers created by the use of health terminologies and jargon, which might have made health messaging abstract, misunderstood, and unappreciated by the targeted public, should be addressed by relevant stakeholders in the broadcast academics, public health professionals, and the industry [15,16].

Condoms and OCPs were the most common contraceptives used among study participants. This is similar to studies from Spain; Dodoma, Tanzania; Botswana; Limpopo, South Africa; and South-South, Nigeria [27,30,39,40,42]. This may be a result of their ready availability and accessibility over the counter in many jurisdictions. The lower proportion of individuals using EC, compared to other contraceptives, in this study was similar to a recent Nigerian Demographic and Health Survey, nationally and in North-Central Nigeria, as well as a higher uptake of EC among unmarried compared to married women [6]. This is despite the high number of sexual encounters, high history of unplanned pregnancies, and a higher unmet need for contraception among this population [6,43]. There is a need for improved sensitization about ECs to stem the high level of unplanned/unwanted pregnancies and continuous risky sexual encounters. Additionally, there is a need to ensure the availability and accessibility of different contraceptive commodities through acceptable means to improve uptake among this population.

The most common reason for contraceptive nonuse was concerns about side effects and health risks among the study population. This is similar to studies among similar populations in Botswana; Pretoria, South Africa; Benin Republic; Limpopo, South Africa; and South-South, Nigeria [11,25,33,40,42]. This might have been driven by personal experiences or information received from significant others such as friends and family members or due to apparent ignorance, even when they have never used one, as seen in many low- and middle-income countries [1]. Thus, there is a need to individualize contraceptive counseling and choice when encountering young people.

This study shows that being a young adult student is a significant predictor of the acquisition of good knowledge. This is similar to national surveys from the United States and a study from the emerging regions of Ethiopia [35,44]. This may be due to less awareness among teenagers and confusing information on contraception online (to which they are more exposed than other age groups) and their limited capacity to filter and process presented information and make appropriate decisions compared to young adults [6,7,45]. Therefore, there is a need for early contraceptive information, communication, and education before the onset of sexual relations to prevent the negative consequences of unguarded sexual and reproductive behaviors.

Being a diploma student (lower degree) was a significant predictor of a good attitude toward contraception. This is converse to most studies where a higher education significantly predicted a good attitude toward contraception [1,35]. Our result may be due to increased information fatigue following information overload that may occur, which might reduce risk perception and increase nonchalant attitude toward contraception. Information received might have been contaminated over the years with disinformation and misinformation to which higher-level degree students might have been exposed to over the years. Thus, information managers and regulators should ensure that the information provided is of high value and an opportunity for updates targeted at a specific population without infringing on human rights. Additionally establishing a consistent, stand-alone, and well-grounded health broadcasting curriculum in the schools of TV studies, journalism, and mass communication might produce an improved attitude toward contraception and help filter out disinformation during undergraduate years, which might be a departure from the current state of health broadcast training in Nigeria [15,16].

This study shows that being off campus was a significant predictor of a good attitude toward contraception. Disaggregation of the study data based on school residence shows that off-campus students were older students, were married, and had higher income and a higher level of knowledge about contraception compared to on-campus students. Similar demography of off-campus students has been reported among undergraduates in the United States [46]. These demographic characteristics are significant predictors of a good attitude toward contraception [35]. Thus, overly restrictive policies on contraceptive access and stigmatization by on-campus health care providers should be addressed to improve contraceptive uptake as needed.

Good knowledge was shown to be a good predictor of a good attitude toward contraception. This is similar to studies from emerging regions in Ethiopia and Botswana [25,35]. Consistent, appropriate, and targeted delivery of contraceptive information, education, and communication through media advocacy will improve the attitude of current and future health broadcasters, editors, and programmers, which will aid their confidence in delivering family planning messaging and programming and further improve contraceptive use among young people and the population in general [16].

Being Plateau indigenous (a conglomeration of about 50 ethnic groups and a majority population in the state) was a significant predictor of contraceptive use. This is similar to studies from similar populations from South-South, Nigeria; Selangor, Malaysia; and the United States where being a member of the majority population is a significant predictor of contraceptive use [26,40,47]. This might be due to disparities in the levels of awareness, knowledge, attitude, and access related to contraception that have been reported in the majority population. In some instances, minority populations may be wary of the government’s intention to limit minority populations and be skeptical about the safety of government-sanctioned contraceptives [47,48]. The minority ethnic differences especially in minority populations should spur health care providers to provide necessary contraceptive education. Innovative counseling approaches could improve women’s ability to make informed decisions. Interventions to reach out to tertiary students, especially those from minority backgrounds, should be instituted in schools to provide information, communication, and education opportunities to male and female students. Since none of the respondents mentioned any reference to sex education in schools [49], there may be a need to review the impact of the current sex education policies in schools on the sexual and reproductive health behavior of young people.

This study shows that being involved in sexual relations is a significant predictor of good contraception practice. This is similar to a study from Kilimanjaro, Tanzania among a similar population [38]. Sexually active individuals see the need to prevent unwanted pregnancy, STIs, and pregnancy-related health risks as they delay marriage to complete education while pursuing sexual relationships [9,10,50]. It also helps girls and women achieve empowerment to live a healthy and economically productive life. Studies have shown that sexually inactive young people often cite their sexual inactivity as a reason for the nonuse of contraception [42]. The health system should, therefore, be well prepared to assist young people who might need a full range of sexual and reproductive health services whenever and wherever they need them without experiencing any form of hardship.

First, the outcome of this study cannot be generalized to all universities, but the status of the college being the only TV studies college in Africa can provide insight into the knowledge, attitudes, and practices related to contraception among future TV professionals. Second, there may also be a social desirability bias as respondents might have underreported sexual behaviors and contraceptive use. This was minimized by ensuring confidentiality, anonymity, and privacy during and after the study. Third, the study is gender biased, as the study population comprised females only. Though male involvement is a great goal in achieving optimal sexual and reproductive health, females experience more reproductive health issues, have less autonomy over life choices and decisions, are exposed to more stigma while accessing contraceptive commodities, have a higher incidence of STIs, and have a higher incidence of child marriages compared to their male counterparts of similar age. All these adverse inequalities cause young females to experience higher consequential adverse outcomes, especially in low- and middle-income countries, and the need for more studies on their sexual and reproductive health [8].

Female undergraduate NTA TV College students in this study had relatively low levels of good knowledge, attitudes, and practices related to contraception. There is a need for an appropriate and consistent awareness campaign via acceptable media and curricular improvement among TV studies undergraduate students to improve their current knowledge, attitudes, and utilization of contraception. Parent-child communication should be encouraged and supported to improve contraceptive knowledge, attitude, and practice, as the family is the first educational institution in the life of children as they prepare to face the world. There is also a need to evaluate and improve the current comprehensive sex education in many sub-Saharan African countries to have more robust training for young people on sexual and reproductive health as early as possible. There is an urgent need to reform current advocacy efforts, sexual and reproductive health services, and policies to improve contraceptive knowledge, attitudes, and use among young people.

## Supplementary material

10.2196/56135 Multimedia Appendix 1 Questionnaire.

## Abbreviations

aOR: adjusted odds ratio
EC: emergency contraception
OCP: oral contraceptive pill
OND: Ordinary National Diploma
STI: sexually transmitted infection
