# Supplementary material for: Levels and Predictors of Knowledge, Attitudes, and Practices Regarding Contraception Among Female TV Studies Undergraduates in Nigeria: Cross-Sectional Study
Source: JMIRx Med. 2025 May 8;6:e56135. doi: 10.2196/56135 (PMC12080006; doi:10.2196/56135)
Supplement: Multimedia Appendix 1 [file xmed-v6-e56135-s001.docx]

**QUESTIONNAIRE**

**KNOWLEDGE, ATTITUDE AND PRACTICE OF CONTRACEPTION AMONG FEMALE TV UNDERGRADUATES IN NIGERIA**

**SECTION A: SOCIODEMOGRAFIC DATA**

1. Age   [        ]

2. Marital status (a) Single [ ] (b) married[ ] (C) separated[ ] (d) divorced [ ] (e) widowed [   ]

3. Religion (a) Christianity (b) Islam (c)  traditional (d) others : please specify……………..

4. Ethnicity (a) plateau indigenous (b) plateau non indigenous

5. Program (a) Diploma (b) Degree

6. Level in school (a) OND 1 (b) OND 2(c)100(d) 200(e) 300(f) 400

7. Home residence…………………..

8. School residence (a) campus (b) off campus

9. Department……………………..

10. Monthly allowance.........................

**SECTION B: KNOWELEDGE OF CONTRACEPTION**

| **QUESTIONS** | **Yes** | **No** | **I don’t know** |
| --- | --- | --- | --- |
| **1. Have you ever heard of contraceptive?** |  |  |  |
| **2. Birth control pills are effective even if a woman misses taking them for two or three days in a row.** |  |  |  |
| **3. Female sterilization is one way to avoid pregnancy.** |  |  |  |
| **4. Health education is important for women who want to use contraception** |  |  |  |
| **5. Contraceptive pills guarantee 100% protection.** |  |  |  |
| **6.6. Condoms  does not prevent STIs.** |  |  |  |
| **7.Common side effects of contraceptive pills include mood swings and weight gain.** |  |  |  |
| **8. There is an increased risk of breast cancer in women taking estrogen-containing contraceptives** |  |  |  |
| **9. Women using the birth control shot (Depo Provera) must get an injection every three months.** |  |  |  |
| **10. If a woman is having side effects of one kind of contraceptive pill, switching to another type might help.** |  |  |  |
| **11. Using both a condom and the pill is considered to be a very effective** |  |  |  |
| **12.Using the pill increases a woman's risk of cervical cancer.** |  |  |  |

**13. Where do you get information on contraceptives**

**(a). family (b) friends (C) print media [ newspaper, magazines]. (d)Internet [Google, social media] (e) Broadcast media [Tv , radio (f) if others, specify…………………..**

**SECTION C:  ATTITUDE ABOUT CONTRACEPTIVE**

| **QUESTIONS** | **Strongly agree** | **Agree** | **Neutral** | **disagree** | **Strongly disagree** |
| --- | --- | --- | --- | --- | --- |
| **1. Contraceptives should be used to limit my number of children.** |  |  |  |  |  |
| **2. Contraceptives should be used to increase the time interval**  **between my childbirths** |  |  |  |  |  |
| **3. Spacing will allow a child to be healthier.** |  |  |  |  |  |
| **4. The ideal age of having a first child is 20-30** |  |  |  |  |  |
| **5. The ideal number of children should be between 3-5** |  |  |  |  |  |
| **6.Contraceptives provide a sense of safety.** |  |  |  |  |  |
| **7. The method of contraception I am using is adequate.** |  |  |  |  |  |
| **8. Contraceptives benefit males too.** |  |  |  |  |  |
| **9. Discussion about contraception with spouse is embarrassing.** |  |  |  |  |  |
| **10. My husband does not approve my use of contraceptives.** |  |  |  |  |  |
| **11. Contraceptive methods can protect the health of family and community.** |  |  |  |  |  |
| **12. Religious beliefs can prevent women from using**  **contraceptives.** |  |  |  |  |  |
| **13. Cultural beliefs can prevent women from using contraceptives.** |  |  |  |  |  |
| **14.. Husband's objections to contraceptives can prevent women**  **from using contraceptives** |  |  |  |  |  |
| **15. Change in male attitudes on contraceptives may improve**  **contraceptive use** |  |  |  |  |  |

**16. Why do you think you will not use contraceptives?.......................**

**SECTION D: PRACTICE OF CONTRACEPTION.**

| **QUESTIONS** | **Always** | **Usually** | **sometimes** | **seldom** | **Never** |
| --- | --- | --- | --- | --- | --- |
| **1. How often do you visit a health centre for family planning services?** |  |  |  |  |  |
| **2. Do you use contraceptives to prevent unplanned pregnancy?** |  |  |  |  |  |
| **3. Have you ever had any unplanned pregnancy due to lack of contraceptive use?** |  |  |  |  |  |
| **4. Do you use contraceptives every time when you do not intend to get pregnant?** |  |  |  |  |  |
| **5. I use different types of contraceptives.** |  |  |  |  |  |
| **6. My current method of contraceptive changes from time to time** |  |  |  |  |  |
| **7. Do you practice any traditional contraceptive methods including**  **withdrawal, infertility period, herbal and breast feeding if you were**  **not using any contraceptives?** |  |  |  |  |  |

**8. Have used or currently using contraceptives? If yes which one, multiple choice**

**(a)condom (b)oral contraceptive pills(c) intrauterine contraceptive device (d) injectable (e)others, specify**
